# Development of a 3D immersive videogame to improve arm-postural coordination in patients with TBI

**DOI:** 10.1186/1743-0003-8-61

**Published:** 2011-10-31

**Authors:** Ksenia I Ustinova, Wesley A Leonard, Nicholas D Cassavaugh, Christopher D Ingersoll

**Affiliations:** 1 The Herbert H. and Grace A. Dow College of Health Professions, Central Michigan University, MI, USA; 2 Center for Driver Evaluation, Education and Research and Department of Psychology, Central Michigan University, MI, USA

**Keywords:** virtual reality, motor rehabilitation, postural control, brain injury

## Abstract

**Background:**

Traumatic brain injury (TBI) disrupts the central and executive mechanisms of arm(s) and postural (trunk and legs) coordination. To address these issues, we developed a 3D immersive videogame-- Octopus. The game was developed using the basic principles of videogame design and previous experience of using videogames for rehabilitation of patients with acquired brain injuries. Unlike many other custom-designed virtual environments, Octopus included an actual gaming component with a system of multiple rewards, making the game challenging, competitive, motivating and fun. Effect of a short-term practice with the Octopus game on arm-postural coordination in patients with TBI was tested.

**Methods:**

The game was developed using WorldViz Vizard software, integrated with the Qualysis system for motion analysis. Avatars of the participant's hands precisely reproducing the real-time kinematic patterns were synchronized with the simulated environment, presented in the first person 3D view on an 82-inch DLP screen. 13 individuals with mild-to-moderate manifestations of TBI participated in the study. While standing in front of the screen, the participants interacted with a computer-generated environment by popping bubbles blown by the Octopus. The bubbles followed a specific trajectory. Interception of the bubbles with the left or right hand avatar allowed flexible use of the postural segments for balance maintenance and arm transport. All participants practiced ten 90-s gaming trials during a single session, followed by a retention test. Arm-postural coordination was analysed using principal component analysis.

**Results:**

As a result of the short-term practice, the participants improved in game performance, arm movement time, and precision. Improvements were achieved mostly by adapting efficient arm-postural coordination strategies. Of the 13 participants, 10 showed an immediate increase in arm forward reach and single-leg stance time.

**Conclusion:**

These results support the feasibility of using the custom-made 3D game for retraining of arm-postural coordination disrupted as a result of TBI.

## Background

Approximately 3.2 million Americans live with long-term disability following traumatic brain injury (TBI) [1]. The majority of TBI survivors present with disrupted central and executive mechanisms underlying arm and postural (trunk and legs) coordination [2]. Behaviorally, such disruption limits postural stability when performing arm movements, increases the risk of falling, deteriorates motor skills, and eventually decreases the quality of life of TBI survivors [3-5]. Despite the importance of arm-postural coordination, surprisingly little attention is paid to its restoration by conventional rehabilitation, which generally treats the affected upper extremities, postural control, and gait separately. This lack of attention is related to the complexity of TBI-related coordination deficits, inconsistency of evaluation, difficulties addressing a specific problem with real world coordination tasks, and adapting these tasks to the abilities and needs of TBI survivors. Development of customized virtual reality (VR)-based gaming exercises and their implementation into TBI rehabilitation may solve this problem.

To date numerous custom-made VR applications have been tested and shown to be effective in restoring sensorimotor abilities in patients with acquired brain injuries. Utilizing movements similar to those made in the real world, VR games incorporate elements essential for successful retraining. The games offer practice in various and safe environments, allow manipulation with the timing and precision of object interactions, provide real-time performance feedback, and acknowledge a participant's success relative to his/her abilities [6-10]. Although most positive evidence has been collected in patients with stroke [6,8,11,12], several studies reported the feasibility of VR practice in patients with TBI [13,14]. The potential of VR gaming applications has been verified in this population when assessing cognitive functions [14,15], or retraining functional skills [16,17] and balance [9,18,19].

Despite the high potential of VR games, some gaps remain in their development as rehabilitation tools. Most virtual environments use gaming concepts, which simply simulate arm movements, balance, walking, and cognitive tasks separately, with minimal attention paid to restoration of specific motor deficits, such as complex whole-body motions. This fact limits the use of VR applications in patients with multiple TBI-related sensorimotor abnormalities. Furthermore, most customized VR paradigms lack actual gaming elements that could make the task challenging, competitive, motivating, and fun. Virtual-reality paradigms with built-in animation scenarios frequently ignore the basic principles of game development, such as the inclusion of a gaming story, conflict, reward system, and level-based increases in difficulty. Most studies reported results of practicing repetitive, stereotyped actions, as for example pointing at a target in a simulated elevator [11] or reaching to grasp simulated objects [12]. Designing virtual environments which implement the principles of game design would advance virtual rehabilitation in neurological populations.

While absent in custom-made virtual environments, the above basic gaming elements are found in popular "off-the-shelf" games offered for example by Nintendo Wii and Sony EyeToy. Unfortunately available as alternative therapeutic tools, off-the-shelf games cannot replace customized tasks because of the inflexible gaming content, absence of strict requirements for defining and monitoring the precision of motor performance, and lack of equivalency to movements performed in the real world [20,21]. For example, all large-amplitude whole-body movements required for playing tennis can be replaced with only one hand manipulating a Wii controller. Such a distorted spatial calibration diminishes the rehabilitation effect of this gaming system.

Considering the drawbacks of existing VR-based applications, we developed a customized videogame (Octopus) for use by patients with TBI. Unlike many other custom-designed virtual environments, Octopus focused on training arm-postural coordination, utilized the basic principles of game design, and included tasks calibrated according to the patient's anatomical features and movement abilities. This paper provides a description of the game design, training protocol, and effect of a short-term gaming practice on the arm and postural coordination of patients with mild-to-moderate TBI. Some results have appeared previously in abstract form [22].

## Methods

### Gaming system

The gaming system consisted of a Dell Mobile Precision M6500 laptop (Intel i7 quad core CPU) with a graphics accelerator (NvidiaQuadro FX 3800 M) integrated with a 6-camera system for motion capture (Qualisys AB, Sweden). Participant interaction with the simulated virtual environment occurred via hand avatars, precisely reproducing the real-time kinematic patterns. The avatars were created with 3 reflective markers (12 mm in diameter) attached to each hand. The movements of the markers were recorded by the Qualisys system for motion analysis at 100 Hz and then synchronized with the VR gaming scenario with minimum delay. The image was projected in 3D format onto an 82-inch screen (1080 p Mitsubishi DLP^® ^TV bundle, RealD) and was viewed by the participant in the first-person view via shutter glasses (RealD Professional CrystalEyes 5). The glasses are emitter free and thereby caused no interference with the infrared signal emitted by the motion capture system. Wearing these light weight glasses (67 grams) posed minimum constraint on participant's movements.

The gaming scenario was developed using WorldViz's Vizard software (WorldViz LLC) with computer graphics created with Alias' Maya package for 3D animation (Maya^®^, Version 7.0.1; Autodesk, Inc.).

### Gaming Scenario

**Figure 1:**
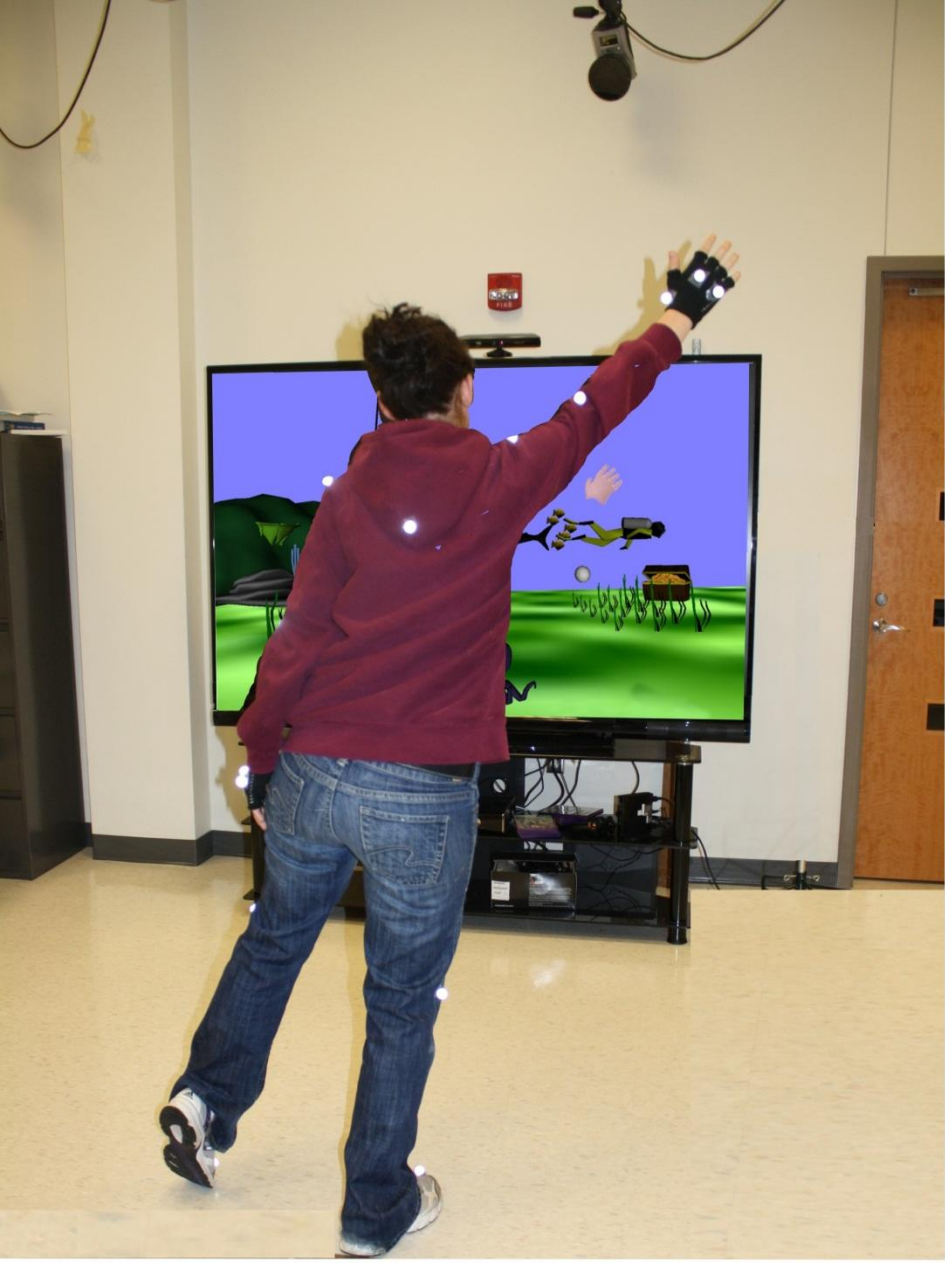
**Experimental setup with subject standing in front of the screen with the Octopus scenario projected**. Image is taken from the seventh gaming trial.

The game is designed to challenge postural stability while reaching to intercept a moving target. In the gaming scenario, all actions occur in an underwater world populated with seaweeds and corals (Figure 1). The main character, Octopus, is located in the middle of the screen. Octopus blows bubbles towards the participant, whose presence in the underwater landscape is indicated by the right and left hand avatars.

**Figure 2:**
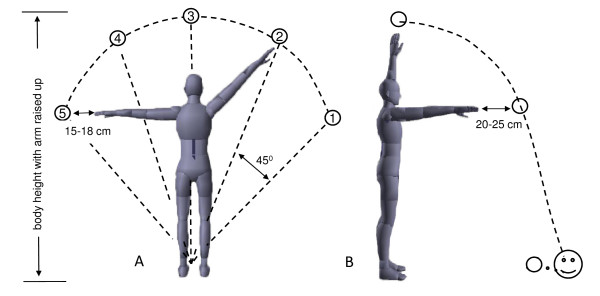
**Calibration of virtual space with bubble in the frontal (A) and sagittal (B) planes**. The bubble trajectories in the frontal plane are numbered from 1 to 5 to simplify description in the text; the octopus location is marked by the smiley in figure 2B.

The gaming task is to reach and pop (intercept) as many bubbles (targets) as possible with the left or right hand. Once launched, each target randomly follows 1 of 5 radial (circular) trajectories (Figure 2A), designed so that the target in the overhead position corresponded to the participant's height with the arm raised up. At shoulder level the target is 25-30 cm beyond the length of the arm, outstretched forward (Figure 2B). This reaching distance is considered as the lower border for norms on the Berg Balance test [20]. This trajectory allows flexibility in the reaching strategy used to intercept the target. When approaching it in a strictly sagittal plane, the participant can reach the target overhead (by maximally extending the arm upwards), at shoulder level (by extending the arm forward), or somewhere between these two critical points. Catching the target at the shoulder level might take less time, but it also causes greater postural displacement, since it requires that the participant lean his/her entire body forward. In the frontal plane, bubbles are aligned along a semicircle, with a 45° interval between trajectories (Figure 2A). The upper target (#3) is within reaching distance, while the lower targets (#1, #5) are 15-18 cm beyond the length of the arm, outstretched to the right or left side. This distance corresponds to norms for healthy individuals on the Multi-Directional Reach Test [23]. Visual representations of the hand and bubble are matched in size.

The gaming session begins with a narration that presents the story of Octopus, provides the gaming instructions, and describes the reward system. In part, the narrative instructs the participant to pop as many bubbles as possible for 90 s without leaving an initial position, losing balance, or taking a step. Each successful bubble trajectory interception is rewarded with points, which accumulate throughout the gaming trial and serve as the criteria of performance success (performance score). The result of the first gaming trial is used to establish a baseline score. Each following trial is rewarded with appearance of an additional character on the Octopus landscape, when the number of bubbles popped was ≥110% of the score on the previous trial. The set of characters includes fish, shark, treasure chest, diver and so on (Figure 1). All the characters appear in particular points of the screen and add some entertaining effect to the game. For example, the fish crosses the screen horizontally at different speeds, the sea horse moves up and down in the left corner of the screen, and the diver is swimming on the right. The ultimate goal is to collect as many characters as possible and get the final reward, an animated Mermaid, who is sitting on the coral reef and waving her tail. All these actions are added to slightly distract participants. The participants were instructed to catch the bubbles, but not the characters. The Octopus blows bubbles regularly every 4*sec *at a speed of 1.5 m/*s*. Successful interception causes the next bubble to appear earlier (immediately after) and thereby increases the bubble flow rate. The maximum possible flow rate is 1/*sec*, but none of our participants reached it. In case of unsuccessful bubble catch, the next bubble follows the regular flow rate. All participants practiced the Octopus game with the same gaming parameters.

#### Participants

The feasibility of the game was tested in 13 individuals (6 males and 7 females, 32 ± 6.7 year old) with chronic mild-to-moderate manifestations of TBI. Table 1 shows the clinical and demographic data for the subjects enrolled in the study.

**Table 1:** Demographic data and clinical scores of patients with TBI

Subject	Age	Sex	Years Since TBI	Arm Dominance	FGA Score	Ataxia Score*	Berg Balance Score				
							
							*Total*	*#6 Forward Reach (cm)*		*#12 Single Leg Stance (s)*	
								*Pre-test*	*Post-test*	*Pre-test*	*Post-test*
S1	41	M	4	R	24	5	45	28	29	16	19
S2	41	M	6	R	20	12	39	23	25	8	10
S3	19	M	2	R	24	11	41	21	17	> 30	> 30
S4	34	F	1.5	R	21	5	48	19	18	5	9
S5	29	F	10	R	29	3	55	24	27	15	25
S6	22	M	4	R	27	5	52	27	29	19	22
S7	38	F	10	R	21	6	55	29	31	3	7
S8	44	F	8	R	27	5	54	25	25	9	10
S9	44	F	10	R	15	9	48	19	23	5	4
S10	38	M	10	L	12	8	49	18	19	8	10
S11	38	M	5	R	27	4	44	32	34	> 30	> 30
S12	33	F	3	L	18	11	43	24	27	> 30	> 30
S13	20	F	2	R	23	9	53	19	24	22	25

*Ataxia scale quantifies the severity of each of the following symptoms: ataxia of gait, stance, upper and lower extremities, dysdiadochokinesis, intention tremor, and dysarthria on a 6-point scale, with grade 0 (symptom is absent) to 5 (unable to perform). All symptom scores are summed with a total score ranging from 1 to 21 corresponding to mild-to-moderate ataxia, and the score 35 indicating the most severe manifestations.

Participants had mild-to-moderate coordination deficits affecting gait, postural control, and upper extremity movements, with clinical test scores ranging as follows: a) 39-55 points on the Berg Balance test [24], with 45 points indicating a high fall risk; b) 12-29 points on the Functional Gait Assessment Test [25], with 22 points indicating a high fall risk; and c) 5-12 points on the Ataxia Test according to Klockgether [26], with 35 points identifying severe ataxia. The participants had low scores (< 10 points) on the Motion Sensitivity Test [27], indicating that no adverse effect (e.g., dizziness, nausea, or disorientation) likely would occur as a result of head or 3D environment motions. Measured as part of the Berg Balance test, the forward reach (item #6) and single-leg stance (item #12) were ranked and included in the total score, and were analysed separately in terms of absolute values (e.g., forward reach in cm and single-leg stance in s, not exceeding 30 s). All participants were able to stand unsupported for at least 2 min. They demonstrated near to full range of motion in upper extremities and had no marked increase in muscle tone. Of the 13 participants, only 3 (#3, #4, #12, Table 1) had mild impairments of arm functions. Arm functions were evaluated with the Arm and Hand section of the Fugl-Meyer stroke assessment scale [28]. The above mentioned participants had scores 62/66; 64/65, and 66/61 for the dominant/non-dominant arms, where a score of 66 corresponds to normal functioning. The participants reported normal stereovision, and normal/corrected visual acuity. The rapid cognitive assessment with the Mini-Mental State Examination scale [29] indicated that no participant exhibited severe abnormalities that might significantly restrict game performance The participants had high scores ranging from 22-30, with ≤ 9 points indicating severe cognitive dysfunction. All participants signed informed consent documents prepared in compliance with the Helsinki Declaration and approved by the Institutional Review Board.

### Training Protocol

Participants practiced the Octopus game 10 times during a single practice session. Each 90-s game included ~20-25 reach-to-pop movements, with a total of 200-250 repetitions per session. To avoid fatigue, a 1-2 min rest period (in standing or sitting position) was allowed between trials. A retention test of 2 gaming trials was administered 30 min after the end of the practice. The 2 retention trials repeated the first and last gaming scenario (with no characters and with the complete number of virtual characters, respectively) of the practice session, with the scores averaged. Before the practice session practice began, a single game trial was introduced to participants to familiarize them with the game content and thereby to reduce a warm up effect. The forward reach and single leg stance items of the Berg Balance test were repeated twice, at the beginning (pre-test; Table 1) and end (post-test) of the gaming session, to evaluate the effects of the short-term practice. On average, each gaming session lasted for ~1 h, including time for the practice itself and rest.

#### Subjective assessment

In order to assess the usability of the game, a 28-item self-report questionnaire based on the work of Qin et al. [30], was administered to all participants. The questionnaire was presented in the format of a 7-point scale, and patients were asked to report their agreement (7 point), disagreement (1 point), or neither agreement or disagreement (4 point) with respect to a number of statements. The questionnaire was designed to assess seven aspects of game satisfaction: control, curiosity, concentration, comprehension, challenge, empathy and motion/fatigue. Some of the items were altered to fit the type of game we used and three questions were added to address the motion/fatigue aspects of the project.

### Data Collection and Analysis

For data analysis, bubble trajectories in the *x, y*, and *z *directions were synchronized with the whole-body movements. These data were recorded by the Qualisys system for motion analysis at 100 Hz, using 30 reflective markers placed on the major bony landmarks. Reaching-to-pop bubble #3 (Figure 2A) with the dominant hand) was analysed kinematically according to the following parameters: arm movement time, arm trajectory curvature, and arm-postural coordination.

The trajectory curvature, analogous to Levin's index of curvature [31], was calculated as the ratio of the actual arm trajectory length to the shortest path to the target. A ratio of 1 indicates no arm deviation from the shortest path, while a ratio of 2 indicates that the arm trajectory was twice as long as the shortest distance between the initial and final arm positions.

The arm-postural coordination was analysed in terms of movement variation and the contribution of body segments, using principal component analysis (PCA) as described by Mah et. al. [32] and modified by Alexandrov et al. [33]. From kinematic data, the angular displacements of 9 body segments (i.e., 2 hands, 2 forearms, 2 upper arms, 1 trunk, and 2 legs) were computed in the sagittal (flexion-extension) plane, typically relative to the sagittal movement of the bubble. The leg, consisting of the thigh and shank, was analysed as a single segment since the angular displacement at the knee joint was minimal for this task.

The vector of temporal variation of the 9 segmental angles φ_i _around their mean values φ_mi _(i = 1,2...8,9) was represented in PCA as a weighted sum of orthogonal and normalized compounds, i.e., a sum of principal components (PCs):

$$\begin{array}{c} \left(\begin{array}{c} \phi_{1}\left(t\right)-\phi_{m1}\\ \phi_{2}\left(t\right)-\phi_{m2}\\ ...\\ \phi_{8}\left(t\right)-\phi_{m8}\\ \phi_{9}\left(t\right)-\phi_{m9} \end{array}\right)=\left(\begin{array}{c} w_{1,1}\\ w_{1,2}\\ ...\\ w_{1,8}\\ w_{1,9} \end{array}\right)∙\xi_{1}\left(t\right)+\left(\begin{array}{c} w_{2,1}\\ w_{2,2}\\ ...\\ w_{2,8}\\ w_{2,9} \end{array}\right)∙\xi_{2}\left(t\right)+...\left(\begin{array}{c} w_{3,1}\\ w_{3,2}\\ ...\\ w_{3,8}\\ w_{3,9} \end{array}\right)∙\xi_{3}\left(t\right) \end{array}$$

where w_ij _was the weight of the segmental angle variation φ_j _in the *i*th PC. Each *i*th PC in the equation above was defined by a vector of 9 constant normalized weights w_ij _(j = 1,2...8,9), called PC loading, and by a corresponding time-dependent scaling factor ξ_i_(t), called PC factor. The PC loading had a positive (or negative) sign when the corresponding segment shifted toward (or away from) the bubble. The PC loading indicated the contribution of each individual segment to the arm-postural coordination, with a mean PC loading > 0.7 being considered significant [33]. The covariance matrix used for the PCA included non-normalized values to enhance the contribution of relatively small segmental displacements. Each movement parameter was measured in a time window between the initial bubble launch and its interception. Performance success was measured as the number of popped bubbles in a 90-s trial. Preliminary individual data were averaged across 13 subjects, each performing 3-4 reaches/trial of the selected bubble with the dominant arm.

Data normality was verified with the Kolmogorov-Smirnov test (p > 0.5). The means were compared using a one-way ANOVA, repeated over eleven times, including practice and retention trials. A paired t-test was used for within-group comparison of the Berg Balance test scores (forward reach and single leg stance) between pre-test and post-test.

## Results

### Game performance

**Figure 3:**
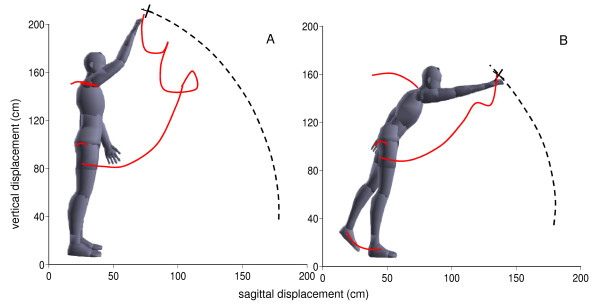
**Trajectories of the arm (hand), trunk (C7), and legs (hip) in sagittal plane during the first (A) and last (B) gaming trials in a representative participant**. Gray model serves as a link between segment trajectories to illustrate body movements. The bubble trajectory is marked by a dashed line. The cross indicates the point of hand/bubble trajectory interception.

While practicing the game, the participants were not instructed on how to move to catch a bubble successfully. The bubble trajectory could be intercepted using different combinations of arm and postural segment displacements. Figure 3 shows the sagittal displacements of the bubble, hand, trunk, and legs (red lines) in a representative participant during the first trial (Figure 3A) and the last trial (Figure 3B). The gray body model in both figures serves as a link between the trajectories and illustrates the participant's movements. His initial attempt to reach the bubble was characterized by a longer and less-accurate hand movement (Figure 3A). The target trajectory (dashed line) was intercepted at an almost overhead position that required minimal postural involvement. By the end of the practice session on the ninth trial (Figure 3B), the hand trajectory became shorter, less curved, and the bubble trajectory was intercepted earlier than on the first trial. To reach the bubble, the participant leaned forward and used his leg to counterbalance the forward body shift. This later strategy revealed the greater involvement of postural segments into arm transport.

**Figure 4:**
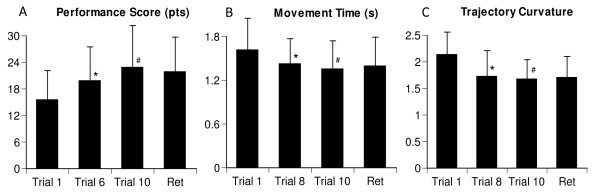
**Means and standard deviations of the performance score (A), hand movement time (B), and trajectory curvature (C) during the first, middle, last, and retention trials**. The middle trial is the trial when significant changes in presented parameter were observed. * identifies significant difference between the first trial and the trial where significant changes occurred; #- identifies significant difference between the first and the last trial.

All participants improved in game performance during the practice session (F_1,10 _= 3.93, p < 0.001), increasing the number of bubbles popped from 15.5 ± 6.72 (mean ± SD) on the first trial to 22.8 ± 10.0 on the last trial. Significant improvement occurred by the sixth trial (Figure 4A, post-hoc p = 0.024) and was retained over a 30-min break to 21.8 ± 7.91 (p = 0.605). Improvement occurred despite the inclusion of additional virtual objects, which appeared after each successful gaming trial and distracted participants' attention. The increased number of popped bubbles through the practice session was proportional to the decreased time of bubble trajectory interception (F_1,10 _= 2.57, p = 0.008, Figure 4B) from 1.61 ± 0.44 s to 1.42 ± 0.35 s on the eighth trial (post-hoc p = 0.034), and to 1.35 ± 0.39 s on the last trial (post-hoc p = 0.010). The movement time measured during the retention test (1.39 ± 0.44 s, p *= *0.076) indicates that no changes occurred after the 30-min retention interval.

A similar decrease in the trajectory curvature (F_1,10 _= 4.15, p = 0.012, Figure 4C) was observed through the gaming session, from initial ratio 2.13 ± 0.43 to 1.72 ± 0.49 (p = 0.041) on the eighth trial with the final mean of 1.67 ± 0.37 on the last trial (p = 0.002), with partial retention of the ratio 1.7 ± 0.39 over the retention interval. Thus the above results indicate that our participants improved on all three parameters characterising game performance. The performance score itself was changed after half of the trials were completed, with two other parameters improved upon completion of about ¾ of trials. The skills were partially retained over a rest interval.

### Arm-postural coordination

**Figure 5:**
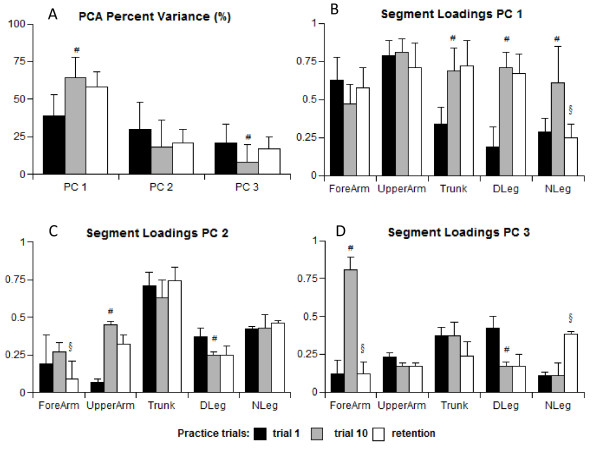
**Means and standard deviations of the percentage of variance explained by the first 3 PCs (A), and segmental loadings of the dominant forearm, upper arm, trunk, dominant and non-dominant legs on the first 3 PCs during the first (B), last (C), and retention (D) trials**. * identifies significant difference between the first trial and the trial where significant changes occurred; #- identifies significant difference between the first and the last trial; §-identifies significant difference between the last and retention trials. The differences are indicated for the PCs loadings coefficients exceeding 0.7.

The movement coordination and relative contribution of different body segments to arm transport were analysed using PCA. About 90% of the variance in the angular displacements of the 9 segments was accounted for by the first 3 PCs (Figure 5A). The amount of variance explained by PC1 was significantly different for reaches-to-pop during the first gaming trial (39% ± 12%) than for those during the last trial (64% ± 14%). The percentage of angular variance associated with PC1 provided evidence of coupling between the segments when popping the bubbles. This coupling was increased by the end of the practice session (F_1,10 _= 2.97, p = 0.003). The first significant change in percentage was observed by the eighth trial (p = 0.023) with a tendency to retain acquired coordination (58% ± 10%) over retention interval.

Performance of the gaming tasks involved the entire body, with 9 body segments (see the Methods section) included into variance clusters. Not all of these segments, however, had a significant impact on movement productions as was determined by the loading coefficients (< 0.7). Figures 5B-D show the PC loadings from the 5 body segments only (dominant forearm and upper arm, trunk, and dominant and non-dominant legs) that contributed significantly to the whole-body movement and had a coefficient exceeding 0.7 at least once throughout the data analysis. The loading coefficients are presented for the first, last, and retention trials. The upper arm loadings in PC1 across the first and last trials indicated that, regardless of the trial, this segment consistently contributed (> 0.7) to the bubble popping and represented the reaching movement itself. The upper arm involvement remained practically unchanged throughout the entire gaming session F_1,10 _= 0.98, p = 0.184). The forearm loading during the last trial was smaller than during the first one (F_1,10 _= 2.45, p = 0.045), probably due to the reduced excursion of the forearm motion relative to the upper arm.

The pattern of loading coefficients for the postural segments (trunk and legs) was markedly different as participants progressed from the beginning to the end of the gaming session. These coefficients were much smaller on PC1 during the first trial, indicating their weak coupling to arm motion. In PC2 and PC3, the postural segment loadings were larger, with trunk motion being coupled (PC2; > 0.7) and the legs contributing insignificantly (PC3; < 0.7). The loadings of the postural segments, on PC1 increased dramatically by the end of practice (F_1,10 _= 1.99, p = 0.031 for the trunk; F_1,10 _= 4.78, p = 0.003 for the leg on the dominant side), signifying that these body parts became greater contributors to the arm transport. Significant changes (p = 0.018) were observed by the eighth trial for the trunk segment and by the ninthh trial for the leg segment (p = 0.021). The leg on the non-dominant side increased its loading (from 0.29 to 0.61; (F_1,10 _= 2.01, p = 0.049) with large inter-subject variation, but did not reach significance (> 0.7). High loadings for the legs and trunk in PC1 on the last trial, combined with the fact that PC1 explained 64% of the variance, indicated that these segments were tightly synchronized with arm motion, forming the synergistic pattern of the whole-body motion. Means of the segmental loadings during the retention test were similar to those recorded during the last gaming trial (post-hoc p > 0.05 for arm, and trunk p ≥ 0.7), except for the leg segment (p = 0.252).

After completing the practice session, 10 of 13 participants displayed improved reaching distances and 9 of 13 increased single-leg stance times (Table 1). Evaluated with the valid [34] and reliable [35] Berg Balance test, the reaching distance was increased on average from 23.6 ± 4.42 to 25.2 ± 5.08 (p = 0.032), and the single-leg stance from 15.4 ± 10.1 to 17.7 ± 9.73 (p = 0.032). Although statistically significant, these changes did not reach a 4-point minimal detectable change threshold [35], indicating the functional improvement to be clinically meaningful. Participants #2 and #9 performed on the pre-test better than on the post-test (Table 1). This might be explained by their fatigue due to practicing.

### Subjective assessment

The descriptive analysis has been used to evaluate the usability of the game with self-report questionnaire data. On the whole patients indicated moderate levels of satisfaction with the game. Results were ambivalent with respect to fatigue. Patients had means of scores 3.42 ± 2.15 points for the survey items "My arms fatigued quickly while playing the game" and 3.46 ± 2.03 points "I got tired quickly while playing the game" where both were near "Neither agree nor disagree". This seems to suggest that the game was subjectively neither too easy nor overly difficult in terms of the physical movements required of patients.

Patients indicated strong interest in the style of the game interface (6.23 ± 1.09), but only moderate interest in the story (3.62 ± 1.98). They reported that they were able to comprehend the game (5.69 ± 1.60), and expressed high agreement with statements about relationships among game characters and events (5.86 ± 1.87). Patients reported high agreement with statements such as "I like the tasks, which are difficult, in the story" (5.50 ± 1.24) and "I feel successful when I overcome the tasks in the game" (6.23 ± 0.93). On the other hand, patients reported moderate to low levels of immersion into the game experience, rating the statement "After playing, it takes a long time for me to return to the real world mentally" with a score of only 2.77 ± 2.05.

## Discussion

Overall, all of our participants benefited from game practice during a single session, displaying improved game performance, arm movement time, trajectory curvature, arm-postural coordination, forward reach, and single-leg stance time. These movement performance changes were partially retained (on average 86%) over the 30-min retention interval.

An increase in the game performance score occurred in most participants at the middle of the gaming session. This first quick improvement was probably associated with understanding of what actions lead to successful performance of the task. Significant changes in movement characteristics, however, were observed much later, upon completion of about ¾ of all reach-to-pop tasks. All improvements most likely were caused by changes in movement strategies. According to the PCA results, a participant's initial effort to pop a bubble began with exploring all possible gaming solutions, making the performance very variable. At that early stage of learning, participants developed an idea of "how to move" that is consistent with classical theories of motor learning [36-39]. Their performance was very variable, characterized by lots of unnecessary motions, interfering rather than facilitating the goal achievement. The later learning followed the "principle of reduction", first described by Skinner in 1938 [39]. All excessive motions and forces were reduced and patients "mastered control of redundant degrees of freedom" [36]. Once movement redundancy was under control, the patients were able to free additional degrees of freedom that were constrained initially. Specifically, later game tasks were performed with greater involvement of postural segments, including the trunk and, in some cases, the legs. This strategy allows catching the target earlier and is used in real life by a majority of experienced goal keepers playing soccer or handball. The goalkeepers do not wait until the ball appears in the goal space, but begin leaning toward it much earlier, often immediately after the ball was forwarded. This facilitates catching the ball before it reaches maximum velocity and allows some time to correct the movement of the arm end-point, in case the ball trajectory was anticipated mistakenly. In our game, the target trajectory remained unchanged. However, the participants intuitively chose early target interception. Moreover, their arm and postural segments began moving in a more coordinated manner, increasing movement efficiency and decreasing movement time of reaches-to-pop. This finding is consistent with the results of Kaminski [40] who, using an example of reaching forward while standing, showed that stronger arm and postural coupling allows faster movement performance.

As unsolicited feedback, one participant reported that the gaming practice gave her an "awareness" of whole-body movements. She expressed amazement that such "forgotten" movements could be made during a gaming session without loss of balance or taking a step. While performing activities of daily living that required arm movements while standing, the participant had previously "stiffened" her body to minimize the risk of falling. Upon completing the gaming session, she reported that she had learned how to control her body and was no longer afraid of instability. Indeed, training the patients in how to gain control, rather than in how to stabilize posture, was an ultimate goal of the gaming practice.

The benefits of the greater involvement of postural segments in arm reach-to-pop movements may be disputable from the movement control standpoint. According to Levin et al. [41] and others, trunk involvement in arm transport during seated reaches is a pathological compensatory strategy in patients after stroke. The authors showed that compensatory trunk movements may improve the performance of the paretic arm initially, but this strategy prevents further long-term recovery of more efficient arm movement patterns [42]. A short-term practice with trunk restraint results in greater reach-to-grasp improvements in patients with chronic stroke than practice without trunk compensatory involvement [43]. The same finding is true for seated activities where balance maintenance is not critical. In contrast, trunk and leg movements are an essential part of activities such as dressing, doing laundry, and cooking when performed in a standing position. For these tasks, gaining control of the arm-postural interaction supports successful and safe performance and cannot be excluded without a detrimental effect.

The usability of the game was evaluated with the questionnaire data. Patients indicated moderate levels of difficulty and immersion in the game, reported that the game was subjectively neither too easy nor overly difficult in terms of the physical movements. They liked the tasks and felt successful when overcoming them. Despite the strong interest in the style of the game interface, participants indicated only moderate interest in the story. This is not surprising as the task was not designed with a particularly compelling story. Based on these self-report data, it seems that the game designers were successful in creating a game which was compelling enough to engage patients in the task and provide challenges without being overly fatiguing. There is room for improvement in terms of immersion into the game and in game narrative.

The short-term effect of practicing Octopus, the partial skill retention over time, and participants' satisfaction provide strong evidence of the feasibility and usability of this type of videogame in patients with TBI. These games potentially can be used to challenge postural stability and regain arm-postural control. In contrast to the previously used VR approaches, Octopus has an algorithm which allows manipulating an amount of postural displacements depending on patient's abilities. This is a novel aspect in design of rehabilitation games which encourages maximal use of available coordination strategies.

### Limitations of the study

The study did not address whether the gaming intervention improved functional outcomes. This was a pre-clinical experiment testing the feasibility of a VR game as a rehabilitative tool, which is a necessary step prior to initiating any type of clinical studies. Longer-term practice in the framework of different types of randomized controlled trials is necessary to assess changes in functional abilities. Another study limitation was enrolment of relatively unimpaired individuals, who had nearly full ranges of motions. We can predict that people with restricted arm movements would utilize postural segments for arm transport to a greater extent, as was shown on example of arm swinging while standing in individuals with stroke [44]. This was not confirmed experimentally yet and will be considered in further research. The study did not include a control group. Thus the results cannot be interpreted in terms of whether our TBI participants approached the level of performance in healthy uninjured individuals. Future study is necessary to address this issue.

## Competing interests

The authors declare that they have no competing interests.

## Authors' contributions

All authors were involved in study design. KIU carried out data collection and analysis, and drafted the manuscript. WAL developed the game. NDC conducted subjective assessment and analysis of the questionnaire data, and participated in writing the manuscript. CDI participated in writing the manuscript.

All authors read and approved the final manuscript.

## References

[B1] Corrigan JD Selassie AW Orman JA The epidemiology of traumatic brain injury J Head Trauma Rehabil 2010 25 72 80 10.1097/HTR.0b013e3181ccc8b4 20234226

[B2] Fredericks C Saladin L Pathophysiology of the Motor Systems 1996 FA Davis: Philadelphia

[B3] Arce FI Katz N Sugarman H The scaling of postural adjustments during bimanual load-lifting in traumatic brain-injured adults Hum Mov Sci 2004 22 749 768 10.1016/j.humov.2003.12.002 15063052

[B4] Dault MC Dugas C Evaluation of a specific balance and coordination program for individuals with a traumatic brain injury Brain Inj 2002 16 231 244 10.1080/02699050110103300 11908477

[B5] Kuhtz-Buschbeck JP Stolze H Gölge M Ritz A Analyses of gait, reaching, and grasping in children after traumatic brain injury Arch Phys Med Rehabil 2003 84 424 430 10.1053/apmr.2003.50017 12638112

[B6] Levin MF Knaut LA Magdalon EC Subramanian S Virtual reality environments to enhance upper limb functional recovery in patients with hemiparesis Stud Health Technol Inform 2009 145 94 108 19592789

[B7] Keshner EA Virtual reality and physical rehabilitation: a new toy or a new research and rehabilitation tool? J Neuroeng Rehabil 2004 3 8 11 10.1186/1743-0003-1-8 PMC546404 15679943

[B8] Holden MK Dyar T Virtual environment training: a new tool for rehabilitation Neurology Report 2002 26 62 71

[B9] Sveistrup H McComas J Thornton M Marshall S Finestone H McCormick A Babulic K Mayhew A Experimental studies of virtual reality-delivered compared to conventional exercise programs for rehabilitation Cyberpsychol Behav 2003 6 243 249 10.1089/109493103322011524 12855079

[B10] Weiss Pl Rand D Katz N Kizony R Video capture virtual reality as a flexible and effective rehabilitation tool J Neuroeng Rehabil 2004 20 1 12 10.1186/1743-0003-1-12 PMC546410 15679949

[B11] Subramanian SK Levin MF Viewing medium affects arm motor performance in 3D virtual environments J Neuroeng Rehabil 2011 30 8 36 10.1186/1743-0003-8-36 PMC3145562 21718542

[B12] Magdalon EC Michaelsen SM Quevedo AA Levin MF Comparison of grasping movements made by healthy subjects in a 3-dimensional immersive virtual versus physical environment Acta Psychol (Amst) 2011 in press 10.1016/j.actpsy.2011.05.015 21684505

[B13] Mumford N Wilson P Virtual reality in acquired brain injury upper limb rehabilitation: Evidence-based evaluation of clinical research Brain Inj 2009 23 179 191 10.1080/02699050802695566 19205954

[B14] Grealy MA Johnson DA Rushton SK Improving cognitive function after brain injury: the use of exercise and virtual reality Arch Phys Med Rehabil 1999 80 661 667 10.1016/S0003-9993(99)90169-7 10378492

[B15] Matheis RJ Schultheis MT Tiersky LA DeLuca J Millis SR Rizzo A Is learning and memory different in a virtual environment? Clin Neuropsychol 2007 21 146 161 10.1080/13854040601100668 17366282

[B16] Christiansen C Abreu B Ottenbacher K Huffman K Masel B Culpepper R Task performance in virtual environments used for cognitive rehabilitation after traumatic brain injury Arch Phys Med Rehabil 1998 79 888 892 10.1016/S0003-9993(98)90083-1 9710158

[B17] Schultheis MT Mourant RR Virtual reality and driving: The road to better assessment for cognitively impaired populations Presence 2001 10 431 439 10.1162/1054746011470271

[B18] Thornton M Marshall S McComas J Finestone H McCormick A Sveistrup H Benefits of activity and virtual reality based balance exercise programmes for adults with traumatic brain injury: perceptions of participants and their caregivers Brain Inj 2005 19 989 1000 10.1080/02699050500109944 16263641

[B19] González-Fernández M Gil-Gómez JA Alcañiz M Noé E Colomer C eBaViR, easy balance virtual rehabilitation system: a study with patients Stud Health Technol Inform 2010 154 61 66 20543271

[B20] Bryanton C Bossé J Brien M McLean J McCormick A Sveistrup H Feasibility, motivation, and selective motor control: virtual reality compared to conventional home exercise in children with cerebral palsy Cyberpsychol Behav 2006 9 123 128 10.1089/cpb.2006.9.123 16640463

[B21] Yavuzer G Senel A Atay MB Stam HJ ''Playstation eyetoy games'' improve upper extremity-related motor functioning in subacute stroke: a randomized controlled clinical trial Eur J Phys Rehabil Med 2008 44 237 244 18469735

[B22] Ustinova KI Ingersoll CD Cassavaugh N Short-term Practice with Customized 3D Immersive Videogame Improves Arm-Postural Coordination in Patients with TBI Proceedings of the Virtual Rehabilitation Conference 2011 Zurich. Switzerland 27 29

[B23] Newton R Validity of the Multi-Directional Reach Test: A Practical Measure for Limits of Stability in Older Adults Journal of Gerontology 2001 56 248 252 10.1093/gerona/56.4.m248 11283199

[B24] Berg KO Wood-Dauphinee SL Williams JI Gayton D Measuring balance in the elderly: preliminary development of an instrument Physiother Can 1989 41 304 311 10.3138/ptc.41.6.304

[B25] Wrisley DM Marchetti GF Kuharsky DK Whitney SL Reliability, internal consistency, and validity of data obtained with the functional gait assessment Phys Ther 2004 84 906 918 15449976

[B26] Klockgether T Schroth G Diener HC Dichgans J Idiopathic cerebellar ataxia of late onset: natural history and MRI morphology J Neurol Neurosurg Psychiatr 1990 53 297 305 10.1136/jnnp.53.4.297 2341843 PMC1014167

[B27] Smith-Wheelock M Shepard NT Telian SA Physical therapy program for vestibular rehabilitation Am J Otology 1991 12 218 225 1882973

[B28] Fugl-Meyer AR Jääskö L Leyman I Olsson S Steglind S The post-stroke hemiplegic patient. I. A method for evaluation of physical performance Scand J Rehab Med 1975 7 13 31 1135616

[B29] Folstein MF Folstein SE McHugh PR ""Mini-mental state". A practical method for grading the cognitive state of patients for the clinician" Journal of psychiatric research 1975 12 3 189 98 10.1016/0022-3956(75)90026-6 1202204

[B30] Qin H Rau PP Salvendy G Measuring Player Immersion in the Computer Game Narrative Int J Hum Comput Interact 2009 25 107 133 10.1080/10447310802546732

[B31] Levin MF Interjoint coordination during pointing movements is disrupted in spastic hemiparesis Brain 1996 119 281 193 10.1093/brain/119.1.281 8624689

[B32] Mah CD Hulliger M Lee RG O'Callaghan IS Quantitative analysis of human movement synergies: constructive pattern analysis for gait J Mot Behav 1994 26 83 102 10.1080/00222895.1994.9941664 15753062

[B33] Alexandrov AV Frolov AA Axial synergies during human upper trunk bending Exp Brain Res 1998 118 210 220 10.1007/s002210050274 9547090

[B34] Tyson SF DeSouza LH Reliability and validity of functional balance tests post stroke Clin Rehabil 2004 18 916 923 10.1191/0269215504cr821oa 15609847

[B35] Newstead A Hinman M Tomberlin J Reliability of the Berg Balance Scale and Balance Master limits of stability tests for individuals with brain injury Journal of Neurologic Physical Therapy 2005 29 18 23 16386157 10.1097/01.npt.0000282258.74325.cf

[B36] Bernstein NA The coordination and regulation of movements 1967 London: Pergamon Press

[B37] Fitts PM Posner MI Human performance 1967 Belmont, CA: Brooks-Cole

[B38] Gentile A Carr J, Sheperd R, Gordon J Skill acquisition: action movement, and neuromotor processes 1987 Movement science: foundation for physical therapy in rehabilitation. Rockville, MD: Aspen

[B39] Skinner BF The behavior of organism: An experimental analysis 1938 New York, Appleton-Century-Crofts

[B40] Kaminski TR The coupling between upper and lower extremity synergies during whole body reaching Gait & Posture 2007 26 256 262 10.1016/j.gaitpost.2006.09.006 17064903

[B41] Levin MF Michaelsen SM Cirstea CM Roby-Brami A Use of the trunk for reaching targets placed within and beyond the reach in adult hemiparesis Exp Brain Res 2002 143 171 180 10.1007/s00221-001-0976-6 11880893

[B42] Cirstea MC Levin MF Improvement of arm movement patterns and endpoint control depends on type of feedback during practice in stroke survivors Neurorehabil Neural Repair 2007 21 398 411 10.1177/1545968306298414 17369514

[B43] Michaelsen SM Levin MF Short-term effects of practice with trunk restraint on reaching movements in patients with chronic stroke: a controlled trial Stroke 2004 35 1914 1919 10.1161/01.STR.0000132569.33572.75 15192250

[B44] Ustinova KI Goussev VM Balasubramaniam R Levin MF Disruption of co-ordination between arm, trunk and centre of pressure displacement in patients with hemiparesis Motor Control 2004 8 139 160 15118199 10.1123/mcj.8.2.139

